# Green synthesis of chitosan nanoparticles, optimization, characterization and antibacterial efficacy against multi drug resistant biofilm-forming *Acinetobacter* *baumannii*

**DOI:** 10.1038/s41598-022-24303-5

**Published:** 2022-11-18

**Authors:** Noura El-Ahmady El-Naggar, Alaa M. Shiha, Hoda Mahrous, A. B. Abeer Mohammed

**Affiliations:** 1 Department of Bioprocess Development, Genetic Engineering and Biotechnology Research Institute, City of Scientific Research and Technological Applications (SRTA-City), New Borg El-Arab City, Alexandria 21934 Egypt; 2 Microbial Biotechnology Department, Genetic Engineering and Biotechnology Research Institute, University of Sadat City, El Sadat City, Egypt; 3 Industrial Biotechnology Department, Genetic Engineering and Biotechnology Research Institute, University of Sadat City, El Sadat City, Egypt

**Keywords:** Nanoparticles, Nanoparticles

## Abstract

Chitosan nanoparticles (CNPs) are promising versatile cationic polymeric nanoparticles, which have received growing interest over last few decades. The biocompatibility, biodegradability, environmental safety and non-toxicity of the chitosan nanoparticles makes it preferred for a wide range of biological applications including agriculture, medical and pharmaceutical fields. In this study, CNPs were biosynthesized by aqueous extract of *Eucalyptus* *globulus* Labill fresh leaves as bio-reductant. Box–Behnken design in 29 experimental runs was used for optimization of different factors affecting the production of CNPs. The maximum yield of CNPs was 9.91 mg/mL at pH of 4.5, chitosan concentration of 1%, incubation time of 60 min and temperature of 50 °C. The crystallinity, particle size and morphology of the biosynthesized CNPs were characterized. The CNPs possess a positively charged surface of 31.1 mV. The SEM images of the CNPs confirms the formation of spherical form with smooth surface. The TEM images show CNPs were spherical in shape and their size range was between 6.92 and 10.10 nm. X-ray diffraction indicates the high degree of CNPs crystallinity. FTIR analysis revealed various functional groups of organic compounds including NH, NH_2_, C–H, C−O, C–N, O–H, C–C, C–OH and C–O–C. The thermogravimetric analysis results revealed that CNPs are thermally stable. The antibacterial activity of CNPs was determined against pathogenic multidrug-resistant bacteria, *Acinetobacter* *baumannii*. The diameters of the inhibition zones were 12, 16 and 30 mm using the concentrations of 12.5, 25 and 50 mg/mL; respectively. When compared to previous studies, the biosynthesized CNPs produced using an aqueous extract of fresh *Eucalyptus* *globulus* Labill leaves have the smallest particle sizes (with a size range between 6.92 and 10.10 nm). Consequently, it is a promising candidate for a diverse range of medical applications and pharmaceutical industries.

## Introduction

Chitosan is a biopolymer that consists of a straight chain and is a cationic polysaccharide. It is produced via the partial deacetylation of chitin. Chitosan has been used in the fabrication of nanoparticles. CNPs are materials with unique physicochemical characteristics, biocompatible, biodegradable, less toxic, easy to prepare and have a wide range of applications in medicine, agricultural and pharmaceutics. Due to their extremely small size, CNPs exhibit interesting interaction and surface characteristics. It is biocompatible and have been used in drug delivery, advanced cancer therapy and biological imaging and diagnosis^1^. It has been indicated by several studies that chitosan nanoparticles could be potential therapeutic agent for viral infections^2^. The extremely positive surface charge of CNPs makes stable nanoparticles that deliver drugs throughout the human body by a variety of mechanisms^3^. CNPs are applied for gene transfer in artificial organs as a controlled-release drug carrier and for immunological prophylaxis. It has been reported that CNPs could act as carriers for active substances that are utilized in hair and skin care products. CNPs were used to provide a prolonged release of hair growth agent, minoxidil sulphate, into hair follicles without dermal exposure^4^. Chitosan nanoparticles were also used as an additive in antimicrobial textiles for healthcare^5^. Chitosan nanoparticles were also used for herbicide delivery for weed eradication^6^, in insecticide^7^, nanofertilizer for balanced nutrition of plants^8^ and fungicide treatment^9^. Chitosan nanoparticles show effective antimicrobial activity against medical pathogens *Escherichia* *coli*, *Klebsiella* *pneumoniae*, *Pseudomonas* *aeruginosa* and *Staphylococcus* *aureus*^10^.

The different methods used for chitosan nanoparticles production include emulsification & crosslinking, precipitation-based methods, ionic gelation, ionic gelation with radical polymerization self-assembly, top–down approach, spray drying and supercritical-CO_2_-assisted solubilization and atomization^11^. The use of chemical and physical methods has many disadvantages which are the use of high-pressure, energy, temperature, toxic chemicals and the large particles size^11–13^. A self-assembled chitosan nanoparticles were prepared in the range 277–731 nm^14^. Nguyen et al.^15^ reported that the mean size of CNPs synthesized using tripolyphosphate (TPP) ionic gelation coupled with spray drying varied between 166 and 1230 nm depending on the chitosan molecular weight and the spray dryer needles diameter. Van et al.^16^ reported that the average size and size distribution of CNPs produced by nano spray dryer were from 300 to 3500 nm and the average size about 1000 nm depending on the size of the spray cap holes. According to the findings of Ha et al.^8^, the size distribution of the CNPs that were synthesized using ionic gelation of TPP and chitosan solution ranged from 300 to 750 nm. Ghormade et al.^17^ reported that the typical size range of nanoparticles that were used for nanoherbicides, nanopesticides and nanofertilizers in agricultural field was between 100 and 500 nm. As a result, there is a critical need to develop environmentally safe strategies for nanoparticles synthesis with ultrafine size.

Green approaches were utilized to produce ultrafine nanoparticles with a size of less than 100 nm, which is a crucial characteristic for a great number of applications in which the specific surface area plays a role^18^. Microorganisms such as bacteria^19^ and fungi^20^ were used for the biosynthesis of nanoparticles. Additionally, secondary metabolites found in plant leaves extracts were used as a reducing agent for nanoparticles biosynthesis^21^. It is claimed that biological agents act as stabilizers, reducers, or both during the nanoparticle formation process^22^. *Eucalyptus* (family, Myrtaceae) is one of the most widely planted genera on the world^23^. Besides essential oils the *Eucalyptus* genus contains; flavonoids (eucalyptin (5-hydroxy-7, 4'-dimethoxy-6, 8- dimethylflavone), triterpenes (ursolic acid), long chain ketones (tritriacontane-16, 18-dione and its 4-hydroxy equivalent), glycosides, acylphloroglucinol derivatives and a combination of many different chemical entities. The leaf waxes are an illustration of the variety of compounds found in *Eucalyptus.*

Antimicrobial drug resistance has progressively developed over the past several decades and is one of the most important challenges since many microbial infections are getting more resistant to currently marketed antimicrobial medications^24–26^. Due to the increasing incidence of pathogenic multidrug-resistant bacteria, the pharmaceutical industry has an urgent need for more rational approaches for the discovery of innovative medications^27^. *Acinetobacter* *baumannii* strains are a common pathogen that can cause severe nosocomial infections acquired in hospitals, particularly in intensive care units. These infections can include bacteremia, pneumonia, and urinary tract infections^28^, skin infections and soft-tissue in patients with burn injuries. Additionally, strains of *Acinetobacter* *baumannii* have the ability to create a biofilm, which is one of the major bacterial pathogens. This is due to the fact that biofilms are resistant to multiple classes of antibiotics including tetracyclines, carbapenems, aminoglycosides, fluoroquinolones, and other extended-spectrum β-lactams^29,30^. Consequently, it is vital to find novel strategies to avoid and treat infections caused by biofilm forming *Acinetobacter* *baumannii* strains.

Biosynthesis of chitosan nanoparticles is affected by various conditions such as temperature, pH, incubation time and chitosan concentration. The statistical design, including the response surface methodology, is efficient for optimization operational parameters. The response surface methodology (RSM) is a set of mathematical and statistical methods for building models, designing experiments, and finding the optimum conditions for optimizing the reaction conditions. There are several advantages for using RSM that includes suitability for multiple factors experiments, less experiment numbers, finding of the best suitable conditions and studying the interaction between the factors ^31–33^.

In the previous studies, the mean size of CNPs synthesized by ionic gelation, nano spray dryer and self-assembly varied between 166 and 3500 nm^8,14–16^. Bekmukhametova et al. ^34^ stated that, it is still challenging to produce a reliable protocol for the fabrication of chitosan nanoparticles in the 200–300 nm range. The chitosan nanoparticles with sizes ranging from 10 to 80 nm shown potential for nanomedicine, biomedical engineering, industrial, and pharmaceutical fields^35^. Therefore, there is a critical need to develop safe strategies for CNPs biosynthesis with ultrafine size for biomedical applications. In the present study, an extract of *Eucalyptus* *globulus* Labill leaves was used to produce ultrafine CNPs with a size range between 6.92 and 10.10 nm. This is a crucial characteristic for many applications where the specific surface area is important.

This study was mainly focused on the green synthesis of chitosan nanoparticles from chitosan solution by using *Eucalyptus* *globulus* Labill leaves extract. The characterizations of the biosynthesized nanoparticles were also performed and the antibacterial activity of the CNPs were evaluated against biofilm forming *Acinetobacter* *baumannii* as a test strain.

## Materials and methods

### Preparation of the plant extract

Fresh *Eucalyptus* *globulus* Labill leaves (Supplementary Fig. S1) were collected from Wadi El Natrun in northwest Egypt (30°21′1.08′′ E longitude and 30°22′39′′ N latitude) 23 m below sea level, and 38 m below the Nile River, Northern West Delta Egypt. Permission was obtained for collection of leaves. The plant was kindly identified by Prof. Dr. Mohamed Fathy Azzazy, head of Surveys of Natural Resources Department, Environmental Studies and Research Institute, University of Sadat City, Egypt. The voucher specimen (*Eucalyptus* *globulus* Labill) has been deposited at the herbarium of Environmental Studies and Research Institute, at University of Sadat City, Egypt. The *Eucalyptus* *globulus* Labill leaves were collected according to institutional, national, and international guidelines and legislation.

The plant leaves were rinsed three times with tap water, followed by a final washing with distilled water to remove any remaining dirt, then chopped into appropriate pieces. For the biosynthesis of chitosan, the *Eucalyptus* *globulus* Labill leaves extract was prepared by mixing 25 g of chopped leaves with one hundred milliliters of distilled water, boiling for ten minutes, and filtering through filter paper. This filtrate represented a 100% *Eucalyptus* *globulus* Labill leaves extract.

### Biosynthesis of chitosan nanoparticles (CNPs)

Chitosan (with purity > 90% and viscosity 60–300 from BIO BASIC INC) got dissolved at 1 percent (w/v) with 1 percent (v/v) acetic acid. The pH was adjusted to 4.8 ± 0.02 with 1 N NaOH. To ensure that the chitosan was entirely dissolved in the solution, it was stirred for twenty-four hours. 10 mL of *Eucalyptus* *globulus* Labill leaves extract was added to 10 mL of the chitosan solution (1:1 v/v). CNPs were obtained by shaking the mixture at 110 rpm for 60 min at 50 °C. After incubation, the CNPs suspension was centrifuged at 10,000×*g* for ten minutes, then freeze dried. The UV/VIS absorbance spectrum of the biosynthesized CNPs was obtained using a double beam spectrophotometer and the highest absorption shown at the wavelength of 295 nm. The concentrations of CNPs (mg/mL) were determined quantitatively based on the standard curve of different concentrations of the lyophilized CNPs (over the concentration range of 1.5–15 mg/mL) with an R^2^ value of 0.9469.

### Box–Behnken design optimization of biosynthesized CNPs

Box–Behnken experimental design^36^ is a response surface method that could be used to obtain maximum response and to observe the interactions among the process factors and the response. To obtain maximum biosynthesis of CNPs, the independent variables including initial pH (A), incubation time (B), chitosan concentration (C), and temperature (D) were coded in three levels (−1, 0, 1). 29 experimental runs using 24 factorial points and five replicates at the central point.

The following second-order polynomial equation is used to fit the RSM experimental results using the response surface regression approach:

1 $$Y={\beta }_{0}+{\sum }_{i}{\beta }_{i}{X}_{i}+{\sum }_{ii}{\beta }_{ii}{X}_{i}^{2}+ {\sum }_{ij}{\beta }_{ij} {X}_{i} {X}_{j}$$

${X}_{i}$ are the coded levels of independent variables, and Y is the predicted response, ${\beta }_{ij}$ is the interaction coefficient, ${\beta }_{i}$ is the linear coefficient, ${\beta }_{ii}$ is the quadratic coefficient, and ${\beta }_{0}$ is the regression coefficient.

### Statistical analysis

The Box–Behnken design was created by Design-Expert software (Version 7.0.0, Stat-Ease, Inc., Minneapolis, MN, USA). Multiple regression analysis was performed on the experimental data to determine the analysis of variance (ANOVA), to determine *P*˗value, *F*-value, and confidence levels. The coefficient of determination (R^2^) and adjusted R^2^. STATISTICA (Version 8, StatSoft, Inc., Tulsa, OK) software was applied to generate three-dimensional surface plots.

## Characterization of chitosan nanoparticles

### UV–visible spectrum

The biosynthesized chitosan nanoparticles were scanned to detect the absorbance peak using an Optizen Pop-UV/Vis spectrophotometer between the wavelengths of 200 and 400 nm.

### Zeta potential of the synthesized chitosan nanoparticles

The zeta potential of the biosynthesized CNPs sample was determined at "Central Laboratories, City of Scientific Research and Technological Applications, Alexandria, Egypt" using a Malvern 3000 Zetasizer Nano ZS, UK”. The concentration of the biosynthesized CNPs suspension was reached to 0.01 weight percent by diluting it with deionized water. The diluted CNPs suspension was first homogenized in a high-speed homogenizer at a speed of 13,000 rpm for 10 min before the analysis, after which it was kept in an ultrasonic bath. The sample was analyzed thrice.

### X-ray diffraction (XRD) pattern

XRD is one of the most essential tools for characterizing the structural features of CNPs. X-ray diffraction measurements were carried out at ambient temperature using a diffractometer type: Bruker D2 Phaser second Gen using a Ni-filtered Cu Kα radiation (λ = 1.54 A°). The generator was running at 10 kV with a current of 30 mA. Data were collected at a scanning rate of 2°/min for 2θ between 0 and 40.

### Differential scanning calorimetry (DSC)

The thermal properties of CNPs were investigated using differential scanning calorimetric (DSC) analysis at the Central Laboratory, City of Scientific Research and Technological Applications, Alexandria, Egypt. Freeze-drying sample of approximately 3.2 mg was used. The sample was analyzed at a flow rate of 30 mL/min under nitrogen atmosphere. The temperatures used during the scan ranged between 25 to 300 °C.

### Thermogravimetric analysis (TGA)

CNPs sample was analyzed using TGA-50H Thermogravimetric analyzer on a sample of approximately 6 mg. The sample was analyzed at a flow rate of 40 mL/min while being subjected to temperatures ranging from room temperature to 500 °C.

### Fourier transform infrared (FTIR) measurements

The FTIR spectroscopy investigation has been carried out in order to investigate the surface characteristics of CNPs. For surface characteristics investigation, sample of CNPs was ground with KBr pellets. The Shimadzu FTIR-8400 S spectrophotometer was used to take the measurements for the CNPs' FTIR spectrum. The scanning range was between 500 and 4500 cm^–1^ with a 1 cm^–1^ resolution.

### SEM and TEM investigations of CNPs samples

Scanning electron microscopy (SEM) investigation was used to detect the surface morphology, size and shape of CNPs. Sample of CNPs coated with gold by using a sputter coater (SPI-Module). The coated CNPs sample was investigated at the Electron Microscope Unit, Faculty of science, Alexandria University, Alexandria, Egypt with SEM “model JEOL-JSM-IT200; at 20 kV”. The CNPs morphology was also investigated by Transmission Electron Microscope (TEM) “JEM-2100 Plus, JEOL Ltd., Japan; at the Central Laboratory, City of Scientific Research and Technological Applications, Alexandria, Egypt”.

Energy dispersive X-ray Spectroscopy (EDX), which obtained using TEM, is often used for determining a sample's elemental composition.

### Bacterial strain, growth conditions and antibacterial activity

Antibacterial activity was tested against multidrug-resistant *Acinetobacter* *baumannii* bacteria isolated from clinical specimens and kindly provided by Mabaret El Asafra Hospital, Alexandria, Egypt. *Acinetobacter* *baumannii* strain is resistant to ticarcillin/clavulanic acid, meropenem, cefepime, imipenem, ceftazidime, piperacillin, piperacillin/ tazobactan, ticarcillin, ciprofloxacin and minocycline. *A.* *baumannii* strain is susceptible to colistin and trimethoprim/sulphamethoxazole drug. A stock culture of *A.* *baumannii* was grown on nutrient agar medium then incubated at 30 °C for 24 h before being stored at 4 °C until use.

The well-diffusion method was used to test the ability of the biosynthesized CNPs to inhibit the growth of multidrug-resistant *Acinetobacter* *baumannii* bacteria using swab inoculation assay method. Bacterial suspension was prepared according to the method of Amini Tapouk et al.^37^. In sterilized Petri dishes, 50 mL of nutrient agar medium was poured and allowed to solidify. After solidifying, the nutrient agar plate surfaces were inoculated by spreading a volume of the bacterial suspension over the nutrient agar plate surface. Then, wells with a diameter of 6 mm were punched aseptically, and a volume of 100 µL of the CNPs were introduced into the well. Petri plates were incubated at 37 °C for 24 h. Following the incubation period, the plates were examined to determine whether or not inhibition zones had developed around the wells. The diameter of the inhibition zone surrounding the well, including the diameter of the well, was measured in millimeters.

### Investigation of chitosan nanoparticles effect on *A. baumannii* cells

The bacterial cells were cultured in nutrient broth medium. After incubation time, the bacterial cells were harvested by centrifuging at 6000×*g* for 10 min and then suspended them in water. The bacterial cells suspended in water were treated with CNPs and incubated at 37 °C for an hour. The residues of treated cells were collected and fixed with formalin-glutaraldehyde fixative in 0.1 M phosphate buffer solution (pH  7.2) at 4 °C for 3 h prior to examination. After treatment, the cell residues were post-fixed in OsO_4_ (osmium tetroxide) at a concentration of 2% in the same buffer for 2 h at 4 °C.

The residues of treated cell were post-fixed in 2% osmium tetroxide (OsO_4_) in the same buffer for 2 h at 4 °C, washed with the buffer, and dehydrated in a graded series of acetones at 4 °C. The residues of treated cells were then embedded in a polymerizing resin before being sectioned to a thickness of 90 Angstroms. Sections should be stained with uranyl acetate for 5 min on the grid cobber, followed by 2 min of lead citrate staining^38^. Transmission electron microscopy (JEOL-JSM-1400 PLUS, Alexandria, Egypt) was used to investigate the effect of CNPs on the cell morphology of the multidrug resistant *A.* *baumannii*.

## Results and discussion

**Figure 1:**
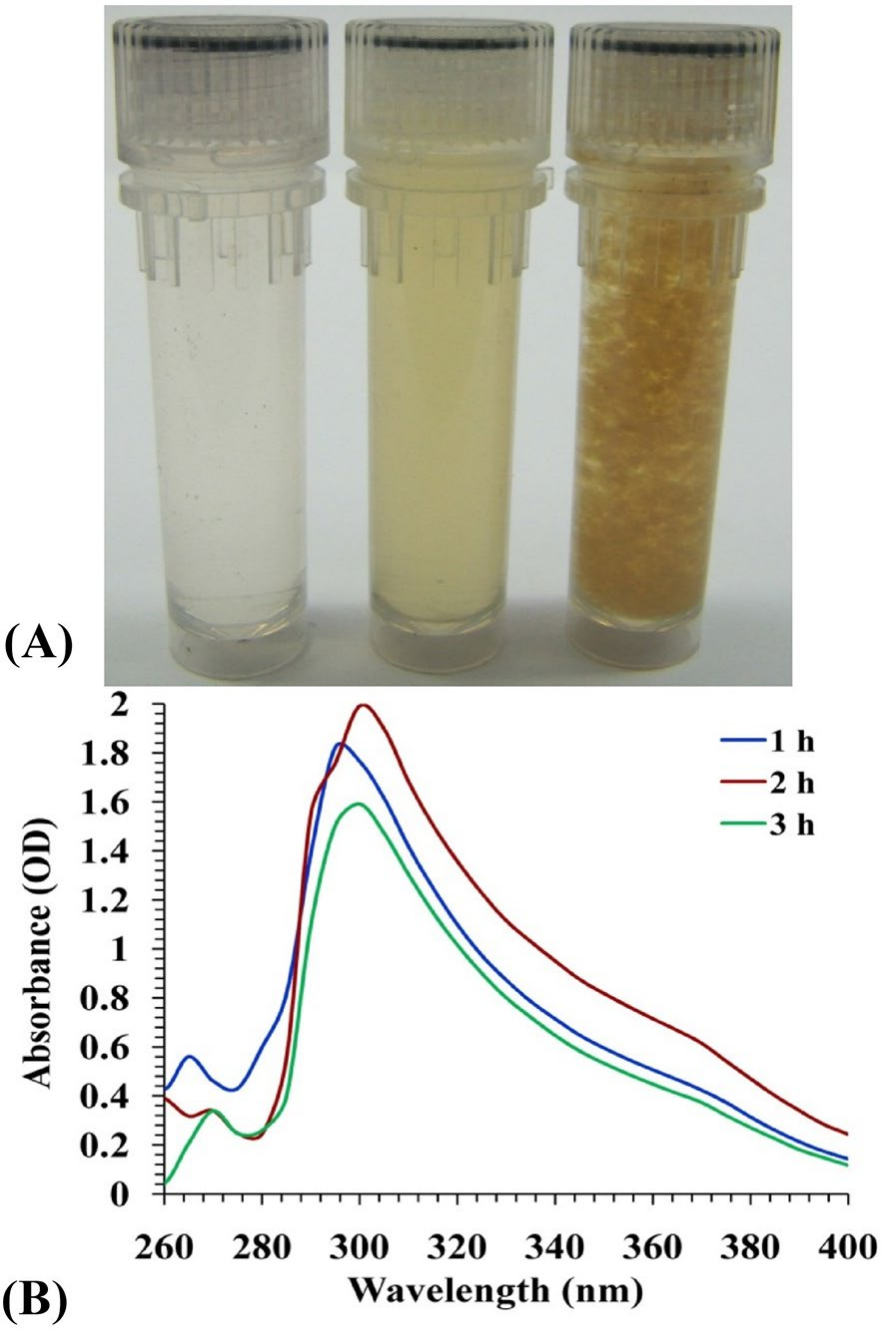
**(A)** Three vials contain (1, Chitosan solution; 2, *Eucalyptus* *globulus* Labill leaves extract; 3, biosynthesized chitosan nanoparticles). **(B)** UV–vis spectrum of chitosan nanoparticles biosynthesised using *Eucalyptus* *globulus* Labill leaves extract.

Many different approaches have been utilized in the synthesis of chitosan nanoparticles. The stability and safety of the CNPs, in addition to the particle size, are factors that should be considered when choosing an acceptable preparation process^39^. In this study, *E.* *citriodora* leaves extract was used for CNPs biosynthesis. Figure 1A shows chitosan solution, leaves’ extract, and the biosynthesized chitosan nanoparticles. The biosynthesized CNPs were analyzed and characterized with the UV/VIS spectral range of 200–400 nm (Fig. 1B). The optical characteristics of CNPs revealed a single peak, and the highest absorbance was measured at a wavelength of 295 nm.

### Optimization of process parameters to maximize chitosan nanoparticles biosynthesis using Box–Behnken design (BBD)

In order to achieve optimal conditions for biosynthesis of chitosan nanoparticles, the Box-Behnken design (BBD) was used. As shown in Table 1, each variable was investigated at three distinct levels (−1, 0, and 1). A total of 29 runs were used to optimize the levels of the selected variables, with 5 runs (runs 15, 17, 21, 25, and 27) at the middle level (center point runs). As indicated in Table 1, the minimum production of CNPs was 3.69 mg/mL in run no. 6 at a pH of 4, an incubation time of 60 min, a chitosan concentration of 0.5%, and a temperature of 50 °C. On the other hand, the maximum production of CNPs was 9.91 mg/mL in run number 15 under the following conditions: pH: 4.5, incubation duration: 60 min, chitosan concentration: 1%, and temperature: 50 °C. Table 1 displays both the actual and predicted results of the biosynthesized CNPs. It can be demonstrated that the experimental results of the biosynthesized CNPs slightly differ from the predicted results.

**Table 1:** Box–Behnken design matrix mean actual and predicted values of chitosan nanoparticles biosynthesis using *Eucalyptus* *globulus* Labill leaves extract.

Std	Run	A	B	C	D	Chitosan nanoparticles (mg/mL)		
						Actual	Predicted	Residuals
5	1	0	0	−1	−1	4.59	4.55	0.04
21	2	0	−1	0	−1	9.36	9.29	0.07
6	3	0	0	1	−1	9.26	9.36	−0.10
15	4	0	−1	1	0	9.51	9.56	−0.05
1	5	−1	−1	0	0	8.71	8.78	−0.07
17	6	−1	0	−1	0	3.69	3.60	0.09
9	7	−1	0	0	−1	8.97	9.04	−0.07
3	8	−1	1	0	0	7.53	7.67	−0.13
8	9	0	0	1	1	8.46	8.33	0.12
12	10	1	0	0	1	8.83	8.83	0.01
4	11	1	1	0	0	8.67	8.43	0.23
23	12	0	−1	0	1	8.16	8.35	−0.18
2	13	1	−1	0	0	8.87	8.58	0.29
10	14	1	0	0	−1	7.96	7.98	−0.02
28	15	0	0	0	0	9.91	9.73	0.18
20	16	1	0	1	0	7.98	8.16	−0.19
27	17	0	0	0	0	9.74	9.73	0.01
14	18	0	1	−1	0	4.63	4.64	−0.01
13	19	0	−1	−1	0	4.64	4.69	−0.05
22	20	0	1	0	−1	8.30	8.22	0.09
25	21	0	0	0	0	9.77	9.73	0.04
24	22	0	1	0	1	8.00	8.16	−0.16
19	23	−1	0	1	0	9.48	9.25	0.23
7	24	0	0	−1	1	4.84	4.58	0.26
29	25	0	0	0	0	9.73	9.73	0.00
16	26	0	1	1	0	8.33	8.34	−0.01
26	27	0	0	0	0	9.50	9.73	−0.23
11	28	−1	0	0	1	7.16	7.20	−0.04
18	29	1	0	−1	0	4.92	5.25	−0.33

Variable	Code	−1	0	1				
Initial pH level	A	4	4.5	5				
Incubation time (min)	B	30	60	90				
Chitosan conc. (%)	C	0.5	1	1.5				
Temperature (ºC)	D	40	50	60				

### The analysis of variance (ANOVA)

The analysis of variance (ANOVA) is shown in Table 2. The determination coefficient (R^2^) was used to check the model's fit. The R^2^ values are a measure of the amount of variance in response values that can be explained by the experimental variables and the interactions between them^40^. The estimated value of R^2^ was 0.9937, and the measure of fit for the model was 99.37%, which indicates that just 0.63% of the variation in the response could not be explained by the model. The R^2^ value is typically in the range of 0 and 1. When the determination coefficient (R^2^) value is close to 1, the applied design is more effective in predicting the response^41^. When the R^2^ value of the regression model is closest to one, it means that the model predicted values are nearly close to the actual values^42^. The adjusted R^2^ value was 0.9873, whereas the predicted R^2^ value was 0.9671. It has been established that the regression model whose R^2^ value is more than 0.9 has a statistically significant correlation^43^. The predicted-R^2^ and Adjusted-R^2^ values must be within 20% of each other, so that we can say that there is a high significance and accuracy of the model and there is a reasonable agreement between them^44^.

**Table 2:** Analysis of variance for chitosan nanoparticles biosynthesis using *Eucalyptus* *globulus* Labill leaves extract as affected by initial pH level, incubation period (min), chitosan concentration (%) and temperature (°C).

Source of variance		Coefficient estimate	Sum of squares	Degrees of freedom	Mean square	*F*-value	*P*-value
Model	Intercept	9.73	99.18	14	7.08	156.77	< 0.0001*
Linear effect	A	0.14	0.24	1	0.24	5.24	0.0381*
	B	−0.32	1.19	1	1.19	26.38	0.0002*
	C	2.14	55.04	1	55.04	1218.01	< 0.0001*
	D	−0.25	0.75	1	0.75	16.59	0.0011*
Interaction effect	AB	0.24	0.23	1	0.23	5.19	0.0389*
	AC	−0.68	1.86	1	1.86	41.21	< 0.0001*
	AD	0.67	1.81	1	1.81	40.04	< 0.0001*
	BC	−0.29	0.35	1	0.35	7.69	0.015*
	BD	0.22	0.20	1	0.20	4.46	0.0532
	CD	−0.26	0.28	1	0.28	6.20	0.0259*
Quadratic effect	A^2^	−0.81	4.20	1	4.20	93.04	< 0.0001*
	B^2^	−0.56	2.05	1	2.05	45.41	< 0.0001*
	C^2^	−2.36	36.17	1	36.17	800.55	< 0.0001*
	D^2^	−0.66	2.86	1	2.86	63.19	< 0.0001*
Error effect	Lack of fit		0.55	10	0.05	2.55	0.1902
	Pure error		0.09	4	0.02		
R^2^	0.9937	Std. dev	0.21				
Adj R^2^	0.9873	Mean	7.91				
Pred R^2^	0.9671	CV %	2.69				
Adeq precision	40.10						

*F* Fishers's function, *P* level of significance, CV coefficient of variation.

*Significant values.

Table 2 shows the *F*-values and *P*-values. *P-*values were calculated to assess the significance of each coefficient and the degree of the mutual interactions between various parameters. As the *P*-values decreased, the significance of the corresponding coefficient increased. In addition, process factors whose confidence levels were greater than or equal to 95 percent and whose *P*-values were less than or equal to 0.05 were considered to have a significant impact on the response^45,46^. The model's *F*-value was 156.77, as well as the *P*-value was less than 0.0001, indicating that the model was highly significant. *P*-value less than 0.05 indicates that the linear coefficients of A (initial pH value), B (incubation time), C (chitosan concentration %), and D (temperature) are significant and they might act as limiting factors with little variations in their values affecting the chitosan nanoparticles biosynthesis rate. A, B, C, and D had *F*-values of 5.24, 26.38, 1218.01, and 16.59, and *P*-values of 0.0381, 0.0002, > 0.0001, and 0.0011, respectively. All quadratic coefficients are significant (*P*-values of > 0.0001), with *F*-values of 93.04, 45.41, 800.55, and 63.19 for the initial pH value, incubation period, chitosan concentration % and temperature (°C), respectively. Moreover, the *P*-values of the coefficients reveal that, among the four factors tested, the interaction between initial pH and temperature (*P*-value < 0.0001) is the strongest, followed by the interaction between initial pH and chitosan concentration percent.

Signs of the coefficients were applied to interpret the data (positive or negative effect on the response)^47^. On the basis of the calculated coefficients (Table 2), linear effects (B, D), mutual interactions (AC, BC and CD), quadratic effects (A^2^, B^2^, C^2^ and D^2^) exerted negative effects on the chitosan nanoparticles biosynthesis. While, positive signs for the linear coefficients of A (initial pH value), and C (chitosan concentration %) indicated a linear effect in the increase in chitosan nanoparticles biosynthesis. The presence of a positive coefficient indicates that there is a synergistic impact being produced by the interactions between two variables. A × B, A × D and B × D affect the chitosan nanoparticles biosynthesis positively. The fact that the interactions between A × B and A × D have statistically significant positive coefficients (*P* < 0.05) suggests that the interactions between these variables contribute significantly to an increase in the biosynthesized chitosan nanoparticles.

The adequate precision value measures the signal-to-noise ratio; a signal-to-noise ratio greater than four is ideal and demonstrates the model's accuracy^48^. The current model has an adequate precision value of 40.10, indicating that it can be used to navigate the design space. The model showed mean, standard deviation and C.V. % (coefficient of variation) values of 7.91, 0.21 and 2.69; respectively (Table 2).

Table 3 show Box–Behnken design fit summary results of CNPs biosynthesis using *Eucalyptus* *globulus* Labill leaves extract. The fit summary used to choose the adequate model for CNPs biosynthesis using *Eucalyptus* *globulus* Labill leaves extract (linear, 2 factors interactions (2FI) or quadratic model). On the basis of the significance of the model terms and the insignificance of the lack of fit tests, the appropriate model is chosen. The fit summary results revealed that adequate model for chitosan nanoparticles biosynthesis is the quadratic model which is significant with *P*-value < 0.0001. Lack of Fit Test for the quadratic model (*F-*value = 2.55 and *P*-value = 0.1902) (Table 3). Furthermore, the model summary statistics for CNPs biosynthesis quadratic model revealed the minimum standard deviation of 0.2126 and the maximum R^2^ of 0.9937, adjusted R^2^ of 0.9873 and the highest predicted R^2^ of 0.9671.

**Table 3:** Fit summary for Box–Behnken design results for chitosan nanoparticles biosynthesis using *Eucalyptus* *globulus* Labill leaves extract as affected by chitosan concentration (%), initial pH level, temperature (°C) and incubation period (min).

Lack of fit tests					
Source	Sum of squares	*Df*	Mean square	*F-*value	*P-*value
Linear	42.51	20	2.13	99.1	0.0002*
2FI	37.77	14	2.70	125.79	0.0001*
Quadratic	0.55	10	0.05	2.55	0.1902

Fit summary					
Source	*P-*value	R^2^	Adjusted R^2^	Predicted R^2^	
Linear	0.0003*	0.5733	0.5021	0.4135	
2FI	0.8852	0.6207	0.41	0.1147	
Quadratic	< 0.0001*	0.9937	0.9873	0.9671	Suggested

*df* degree of freedom, 2FI two factors interaction.

*Significant values.

In order to explore the relationship between independent and dependent variables, a polynomial equation of the second order was used. This equation was used to determine the optimum levels of initial pH (A), incubation time (B), chitosan concentration (C), and temperature (D), as well as the highest CNPs biosynthesis that corresponded to these optimum levels. By using multiple regression analysis to the collected experimental results, the following second-order polynomial equation defining the predicted CNPs biosynthesis (Y) regarding the independent variables (A, B, C, and D) was obtained:

2 $${\text{Y}}\, = \,{9}.{73}\, + \,0.{\text{14 A}}{-\!\!-}0.{\text{32 B}}\, + \,{2}.{\text{14 C}}{-\!\!-}0.{\text{25 D}}\, + \,0.{\text{24 AB}}{-\!\!-}0.{\text{68 AC}}\, + \,0.{\text{67 AD}}{-\!\!-}0.{\text{29 BC}}\, + \,0.{\text{22 BD}}{-\!\!-}0.{\text{26 CD}}{-\!\!-}0.{\text{81A}}^{{2}} {-\!\!-}0.{\text{56 B}}^{{2}} {-\!\!-}{2}.{\text{36 C}}^{{2}} {-\!\!-}0.{\text{66 D}}^{{2}}$$

In which Y is the predicted CNPs biosynthesis, A is the value of the initial pH level, B is the incubation time value, C is the chitosan concentration value and D is value of temperature.

### The three-dimensional (3-D) surface plot

**Figure 2:**
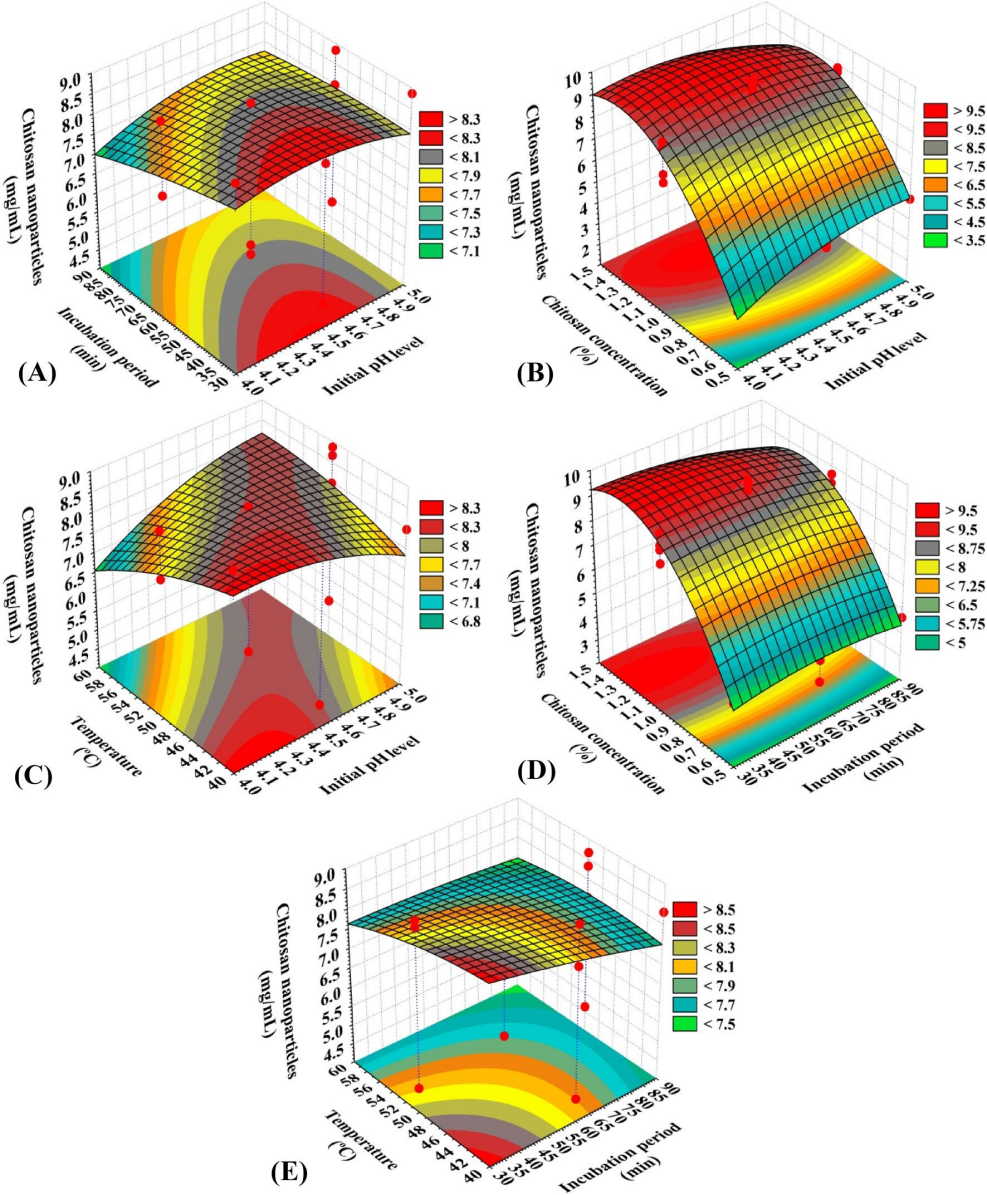
Three-dimensional surface plot for chitosan nanoparticles biosynthesis using *Eucalyptus* *globulus* Labill leaves extract.

The three-dimensional response surface graphs were generated for determination of the pairwise interaction effects between the independent variables (initial pH level, incubation time, chitosan concentration and temperature) as well as the optimal levels of the variables for maximum CNPs biosynthesis (Fig. 2). Kamat et al.^49^ reported that the size, distribution, and number of synthesized particles are controlled by parameters such as temperature, time, and reactant concentration. CNPs biosynthesis (mg/mL) was represented on Z-axis versus two independent factors while the levels of the other two variables were fixed at their center (zero) level to achieve the optimal conditions for maximum CNPs biosynthesis using *Eucalyptus* *globulus* Labill leaves extract. Three-dimensional surface plots of CNPs biosynthesis using *Eucalyptus* *globulus* Labill leaves extract (Fig. 2A–E), illustrates the interacting effects of four evaluated variables.

The 3D surface graphs (Fig. 2A–C) illustrated the effect of initial pH level on CNPs biosynthesis when interacting with the other three variables: incubation time, chitosan concentration % and temperature; respectively. The plots reveal that the CNPs biosynthesis increased by the increasing of initial pH level. Maximum CNPs biosynthesis was obtained toward the center point of initial pH level (around 4.32). Further increase or decrease led to the decrease in the CNPs biosynthesis. Sathiyabama et al*.*^50^ prepared CNPs at a pH of 4.8.

Figure 2A,D,E represents the three-dimensional response surface plots as function of the incubation period on the CNPs biosynthesis when interacting with the other three variables: initial pH level, chitosan concentration % and temperature; respectively. The plots reveal that the CNPs biosynthesis increased by the increasing of incubation period. Maximum CNPs biosynthesis was obtained toward the center point of incubation period (around 41.62 min). Further increase or decrease led to the decrease in the CNPs biosynthesis. Our findings are in agreement with those of El-Naggar et al.^19^ who reported that the highest CNP biosynthesis using *P.* *graveolens* leaves extract was estimated to be 9.73 mg/mL after 57.53 min. Oliveira et al.^51^ found that the incubation period of 12 h is a reasonably suitable time. Saifful and Shahidan^52^ reported that the extension of incubation time to 18 h produced a greater average size of nanoparticles compared with the shorter time (2 h). Sathiyabama et al*.*^50^ prepared CNPs at a chitosan concentration of 0.5% (w/v) with the shorter time of 30 min of magnetic stirring.

On the other hand, Fig. 2B,D depicts the three-dimensional response surface plots as function of the chitosan concentration % on the CNPs biosynthesis when interacting with the other variables: initial pH level and incubation period; respectively. The plots reveal that the CNPs biosynthesis increased as chitosan concentration % increased to the optimal level. Maximum CNPs biosynthesis (9.91 mg/mL) was obtained toward the high level of chitosan concentration % (around 1.29%). Further increase led to the decrease in the CNPs biosynthesis. Vaezifar et al.^53^ found that the optimal initial chitosan concentration was 1.295 mg/mL, chitosan concentration strongly effects the size and formation of the nanoparticles. Handani et al.^54^ reported that the concentration of chitosan has a significant effect on the size of the nanoparticles formed. Kamat et al.^49^ reported that the greatest yield of nanoparticles was obtained with 0.8 mg/mL chitosan. Mahmoud et al.^55^ prepared CNPs at a concentration 2%. Sathiyabama et al*.*^50^ used chitosan concentration of 0.5%.

Figure 2C,E depicts the three-dimensional response surface plots as function of temperature on the CNPs biosynthesis when interacting with the other variables: initial pH level and incubation period; respectively. The plots reveal that the CNPs biosynthesis increased as temperature increased to the optimal level. Maximum CNPs biosynthesis (9.91 mg/mL) was obtained toward the low level of temperature (around 43.18%). The synthesis of nanoparticles decreases as the temperature increases. Handani et al.^54^ results showed that the temperature in ionic gelation method has no effect on the initial size of the nanoparticles formed. The temperature plays a key role in particle development, and shape/size control, it can significantly affect reaction rate, and hence the particle characteristics^56^. Kamat et al.^49^ reported that the greatest yield of chitosan nanoparticles was obtained at 35 °C.

### The model's adequacy

**Figure 3:**
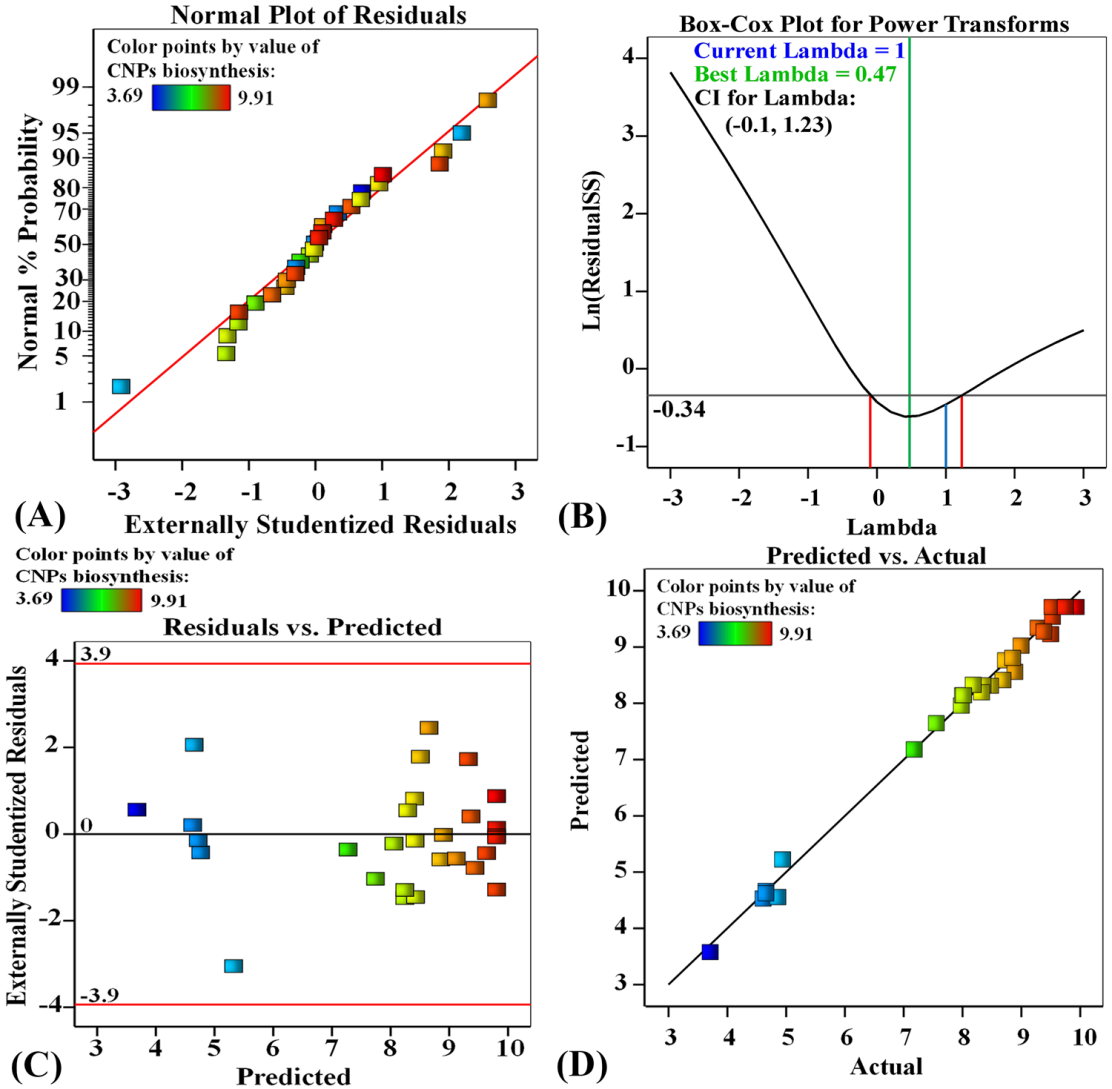
(**A**) Normal probability plot of internally studentized residuals, (**B**) Box–Cox plot of model transformation, (**C**) plot of predicted versus residuals, and (**D**) plot of internally studentized actual values versus predicted values of for chitosan nanoparticles biosynthesis using *Eucalyptus* *globulus* Labill leaves extract.

The normal probability plot (NPP) of the residuals is an important graphical technique represents the residual distribution to check the model's adequacy^57^. In the normal probability of the experimental residuals, as shown in Fig. 3A, the data points collected closely along the straight line, indicating that the residuals follow the normal distribution. The residuals are the variation between the predicted CNPs biosynthesis values by the theoretical model and the experimental values of the CNPs biosynthesis. A small residual value indicates that the model prediction is accurate and the model was well fitted with the experimental results.

Figure 3B represents the Box–Cox plot generated by the model transformation for the biosynthesis of chitosan nanoparticles. As shown in Fig. 3B, the green line represents the best lambda value (λ = 0.47) and the blue line representing the current Lambda (λ = 1). Whereas the red lines represent the minimum and maximum values of the 95% confidence interval, which are -0.1 and 1.23; respectively. The model is in the optimal zone because the blue line of the current Lambda lies between the two vertical red lines. This indicates that the model fits the obtained experimental results well and that no data transformation is required^58^.

In Fig. 3C, the residuals are plotted against the predicted values for the biosynthesis of chitosan nanoparticles. As shown in the graph, the residuals were scattered in a random pattern all around the zero line.

The residuals were scattered equally and randomly above and below the zero line, indicating that the residuals had a constant variance and supporting the model's precision^59^.

Figure 3D depicts a plot of predicted versus actual chitosan nanoparticles biosynthesis. Figure 3D shows all points were collected along the diagonal line, indicating a significant correlation between the theoretical values that were predicted by the model and the actual results of the chitosan nanoparticles biosynthesis which confirms the accuracy of the model^60^.

### The desirability function

The purpose of the desirability function and experimental design was to determine the optimal predicted conditions for maximizing the response. The desirability function values ranged from 0 (undesirable) to 1 (desirable)^61^. The desirability function value is often determined mathematically prior to experimental validation of the optimization process^19^. In this study, the predicted values obtained for the tested variables were as the following: incubation time (41.62 min), temperature (43.18 °C), initial pH level (4.32) and chitosan concentration (1.29%). The maximum predicted value of biosynthesized CNPs was 10.53 mg/mL (Supplementary Fig. S2).

### Characterization of the biosynthesized chitosan nanoparticles by SEM, TEM and EDX

**Figure 4:**
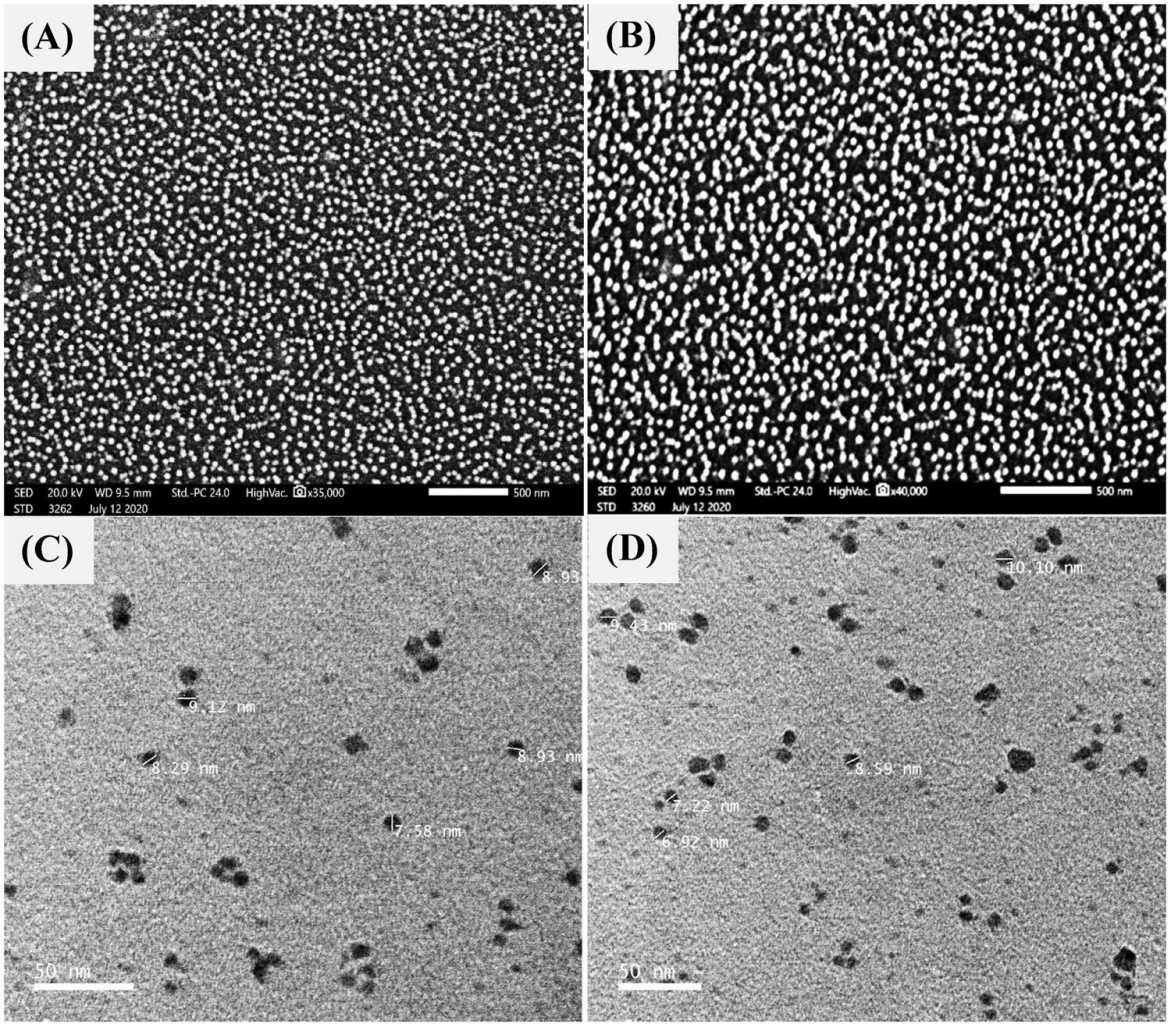
(**A,B**) SEM micrographs, (**C,D**) TEM micrograph of chitosan nanoparticles biosynthesised using *Eucalyptus* *globulus* Labill leaves extract.

Figure 4A–D depicts an investigation of the morphology of biosynthesized chitosan nanoparticles using SEM and TEM, respectively. The morphology of all nanoparticles was relatively homogeneous, with a quite consistent particle size distribution and spherical in shape. The SEM analysis indicates spherical particles with a smooth surface. While, TEM analysis of the obtained biosynthesized chitosan nanoparticles reveals particles ranging in size between 6.92 and 10.10 nm, the particles are spherical. Comparing this study to earlier ones, the biosynthesized CNPs made from an aqueous extract of fresh *Eucalyptus* *globulus* Labill leaves have the smallest particle sizes. The SEM micrograph of chitosan nanoparticles in Wardani et al*.*^62^ study reveals a rough surface morphology and the spheres have diameters around 500 nm. Using FE-SEM, Khanmohammadi et al.^63^ found that the synthesized CNPs have average particle sizes ranging from 33.64 to 74.87 nm. According to the findings of Van et al.^16^, chitosan nanoparticles created by spray drying had a size distribution of 300 to 1500 nm, with an average size of 420 nm, between 500 and 2500 nm, with an average size of 750 nm and the chitosan nanoparticles ranged in size from 700 to 3500 nm, with a mean size of 970 nm, depending on the hole diameter of the spray cap. Bodnar et al.^64^ reported that the range of the TEM-measured particle size was 60 to 280 nm. In Dudhani et al.^65^ study, the chitosan nanoparticles size was 110 ± 5 nm. Zhang et al.^66^ prepared chitosan nanoparticles with size range between 100 and 400 nm.

**Figure 5:**
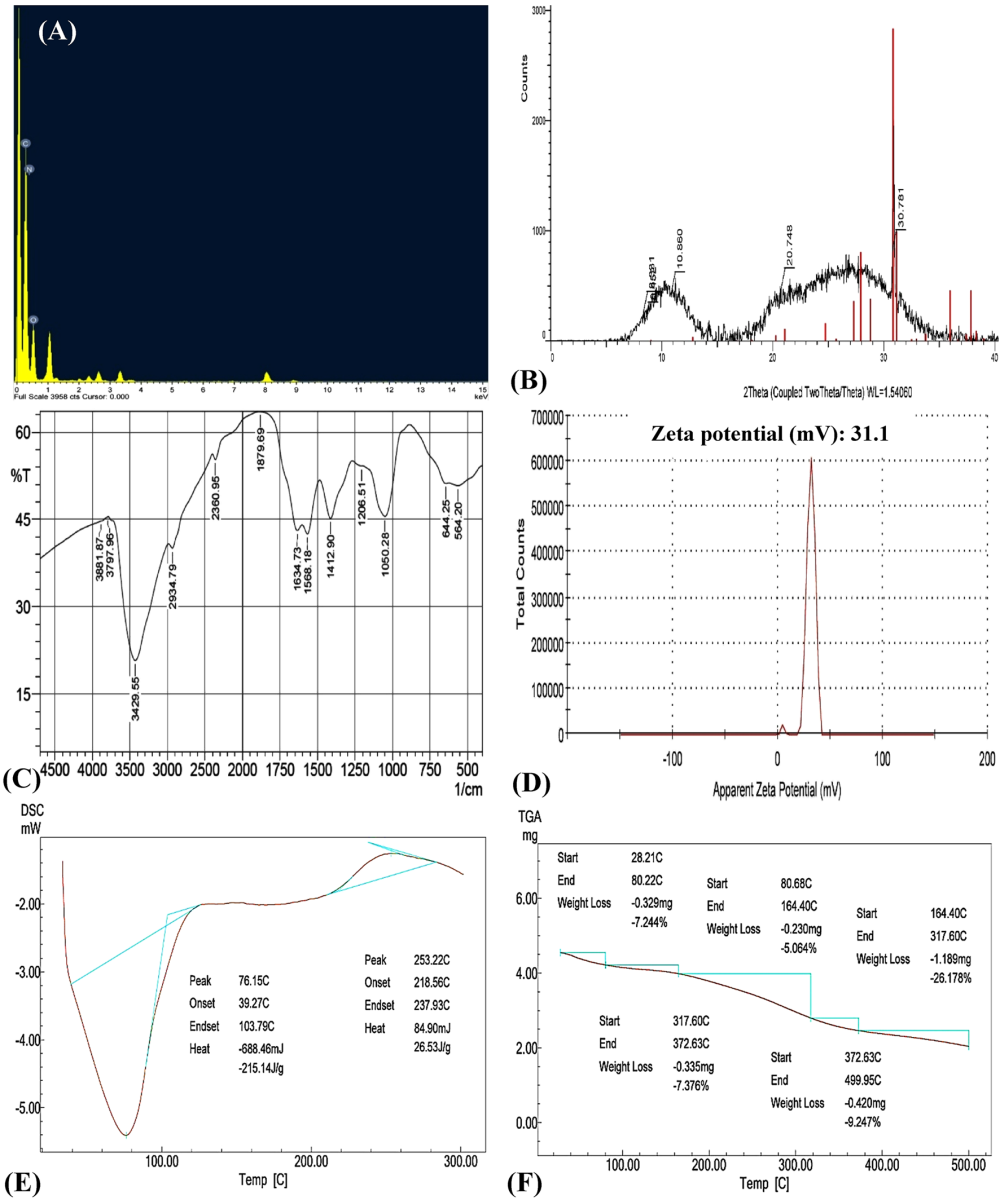
(**A**) EDX, (**B**) XRD, (**C**) FTIR, (**D**) zeta potential, (**E**) DSC and (**F**) TGA analyses of chitosan nanoparticles biosynthesised using *Eucalyptus* *globulus* Labill leaves extract.

The EDX spectrum analysis of the biosynthesized chitosan nanoparticles detected the presence of: carbon (C), oxygen (O) and nitrogen (N), as main elements in chitosan nanoparticles as shown in Fig. 5A.

#### X-ray diffraction (XRD)

An X-ray diffraction pattern was used to recognize the crystal phases of the materials. X-ray diffraction was used to detect the crystallinity of CNPs as shown in Fig. 5 B. The XRD pattern of the dried CNPs was recorded at angles within the range of 10°–40° (2θ) with time per step 132 s, generator tension of 30 kV, generator current of 10 mA, and temperature of 23.7 °C. The XRD pattern of CNPs sample showed three distinctive peaks at 2*θ* which were at 10.86, 20.74 and 30.78° (Fig. 5B) indicating a shift from the normal chitosan peaks. The crystalline structure of chitosan nanoparticles was demonstrated by XRD patterns that displayed strong peak at angle of 20.74° (Fig. 5B).

Similar results were obtained by Rasaee et al.^67^, they reported that the CNPs showed a weak diffraction characteristic peak at 2θ = 10° and a strong diffraction characteristic peak at 2θ = 20°, revealing the high degree of chitosan crystallinity. The spectrum exhibits a large diffraction peak at 2θ = 20°, which was identified as the predominant diffraction peak of (110) chitosan^68^. In the XRD diffraction patterns of the chitosan and chitosan nanoparticles, the diffraction peaks at 2*θ* of 10.18° and 20.26° were observed. Each of these diffraction peaks is a reflection of the (020) hydrated crystalline structure and (110) crystalline structure of anhydrous α-chitin^69^. This indicates the presence of a crystalline phase in the synthesized chitosan nanoparticles^69^. Chitosan's diffraction pattern has peaks at 2*θ* = 9.28° and 20.18°, showing its crystalline form II and a broad band was seen at 2*θ* = 30°^10^. Peaks seen at 2*θ* = 20° and 2*θ* = 10° are typical of chitosan^70^. The diffraction peak around 2*θ* ≈ 30° is indicative of residual chitin in commercial chitosan^13^. On the other hand, Olajire et al.^71^ reported that the peak near 2*θ* = 30º might be an indication of the presence of some inorganic materials that are left behind in the chitosan sample. The XRD pattern of the biosynthesised CNPs showed no peak at 2θ = 16° which is the characteristic feature corresponding to the amorphous structure^72^.

### Fourier transforms infrared (FTIR) measurements

FTIR analysis is a powerful tool revealed various functional groups of organic compounds. Figure 5C and Table 4 shows the FTIR spectrum of CNPs in 4000–500 cm^−1^ range for analysis of functional groups present in their structure. FTIR spectrum has several peaks, the first at 564 cm^−1^ which represents out-of-plane bending NH and out-of-plane bending C−O^73^. The absorption peaks at 644 cm^−1^ attributed to the vibration of –C≡CH^74^. The peaks between 1000 and 1050 cm^−1^ refers to the stretching vibrations of C–OH and C–O–C^75^. Peaks at 1265–1064 cm^−1^ are related to in-plane O–H bending of aromatic compounds, this functional group due to the presence of flavones, terpenoids and polysaccharides compounds in the aqueous leaves extract^76^. The peak at 1412 cm^−1^ represents C–N stretching vibrations (amide III band)^77^. The peak at 1568 cm^−1^ represents –NH_2_ bending vibration^78^. The peak at 1634 cm^−1^ refers to amide I group^79^. The C–O and C–C stretching absorbance peak at 1879 cm^−1^ is related to the release of gaseous compounds that contain aldehyde or ketone or organic acid or alkene groups^80^. The peak at 2360 cm^−1^ is due to CO_2_ bending vibrations^81^. The peak at 2934 cm^−1^ is attributed to aromatic C-H bending vibration^82^. The peak at 3429 cm^−1^ is resulting from O–H stretching^83^. The observed peaks between 3400 and 3800 cm^−1^ are attributed to the O–H, NH_2_ bending vibration, and intramolecular hydrogen bonding^84^. O–H stretching in carboxylic acids is responsible for the peak at 3881 cm^−1^^85^.

**Table 4:** FTIR peaks of biosynthesized chitosan nanoparticles with the annotations and references of each peak.

No	Wave no. (cm^−1^)	Annotations	References
1	564	Out-of-plane bending NH, out-of-plane bending C−O	Varma and Vasudevan^73^
2	644	–C≡CH	Tan et al.^74^
3	1050	C–OH/C–O–C bonds	Vasilev et al.^75^
4	1206	In-plane O–H bending of aromatic compounds	Olajire et al.^76^
5	1412	C–N (amide III band)	Uzun and Topal^77^
6	1568	–NH_2_	Rajam et al*.*^78^
7	1634	Amide I group	Azeez, et al.^79^
8	1879	Release of gaseous compounds	Liu et al.^80^
9	2360	CO_2_	Praffulla et al.^81^
10	2934	Aromatic C–H stretching	Kunasekaran, et al.^82^
11	3429	O–H stretching	Kumirska, et al.^83^
12	3797	O–H, NH_2_	Damiri et al.^84^
13	3881	O–H stretching in carboxylic acid	Anandalakshmi et al.^85^

### Zeta potential

The zeta potential value was used to estimate the surface charge and thus the stability of the synthesized nanoparticles. In this study; the zeta potential value on the CNPs surface was + 31.1 mV indicating that the CNPs have high stability due to higher electrostatic repulsion (Fig. 5D). Kheiri et al.^86^ reported that zeta potentials of the nanoparticles formed were positive due to residual protonated amine groups. Despite the fact that the suspension is physically stable, Muller et al.^87^ and Manikandan & Sathiyabama^88^ mentioned that a zeta potential of at least ± 30 mV is necessary as minimum for a NPs suspension to be stabilized by principally by electrostatic repulsion. If the zeta potential is smaller than + 30 mV, this indicates that the CNPs have less stability due to lower electrostatic repulsion^12^. CNPs have a positive zeta potential, which indicates that they have a charge. According to the findings of Khan et al.^89^, Raza & Anwar^90^ and Asal et al.^91^, the zeta potential on the surface of CNPs was determined to be + 31 ± 3.14, + 31.3 and + 31 ± 2.2 mV; respectively. On the other hand, Qi et al.^92^ reported that the surfaces of chitosan nanoparticles had a positive charge around 51 mV.

### Differential scanning calorimetry (DSC)

The differential scanning calorimeter, or DSC, is a frequently used thermal analytical tool that can assist in understanding the thermal behavior of polymers^93^. The DSC thermogram of CNPs showed two bands, which had typical polysaccharide thermal features. The first was an endothermic wide band corresponding to polymeric dehydration ranged from 39.27 to 103.79 °C. The second thermal band was polymeric degradation, causing an exothermic band extending from 218 to 237 °C as shown in Fig. 5E. Feyzioglu and Tornuk^94^ reported that CNPs revealed an endothermic peak at 120.2ºC and an exothermic peak at 266 °C, which indicates water evaporation and decomposition of the NPs, respectively. Also, Vijayalakshmi et al.^95^ described a wide endothermic peak achieved under 80 °C is outstanding for the removal of absorbed water.

### Thermogravimetric analysis (TGA)

TGA is a thermal analysis technique that detects changes in chemical and physical characteristics of the materials as a function of growing temperature or as a function of time^96^. A thermogravimetric analyzer, model TGA-50H, was used to determine changes in the thermal characteristics of biosynthesized CNPs sample of about 6 mg. At a flow rate of 40 mL/min, the sample was scanned at temperatures ranging from room temperature to 500 °C. The TGA of CNPs is characterized by the presence of five degradation stages (Fig. 5F) at 28.21–80.22 °C, 80.68–164.4 °C, 164.4–317.60 °C, 317.6–372.63 °C, and 372.63–499.95 °C, showing percentage loss values equal to 7.244%, 5.064%, 26.178%, 7.376%, and 9.247%, respectively. These weight losses indicated partial thermal disintegration of CNPs. At heating temperature (500 °C), the total loss was 55.1%^57^. According to Sivakami et al.^97^ findings, the weight loss that occurs between 50 and 150 °C is caused by the evaporation of water, whereas the weight loss that occurs between 200 and 350 °C is due to the heat degradation of chitosan nanoparticles.

On the other hand, Morsy et al.^98^ reported that the TGA diagram of chitosan nanoparticles showed the initial weight loss of 8.56% at 100 °C. This loss is related to the evaporation of intra and inter-molecular moisture in the CNPs. When heated at 200 °C, CNPs had a weight loss of 11.96%, which is consistent with the thermal decomposition of functional groups, such as OH, located on the chitosan backbone. The heat degradation of the chitosan backbone was responsible for the weight loss of 29.17% to 55.38% that occurred between 300 and 500 °C.

### Antibacterial activity of CNPs of against multi-drug resistant *A. baumannii*

**Figure 6:**
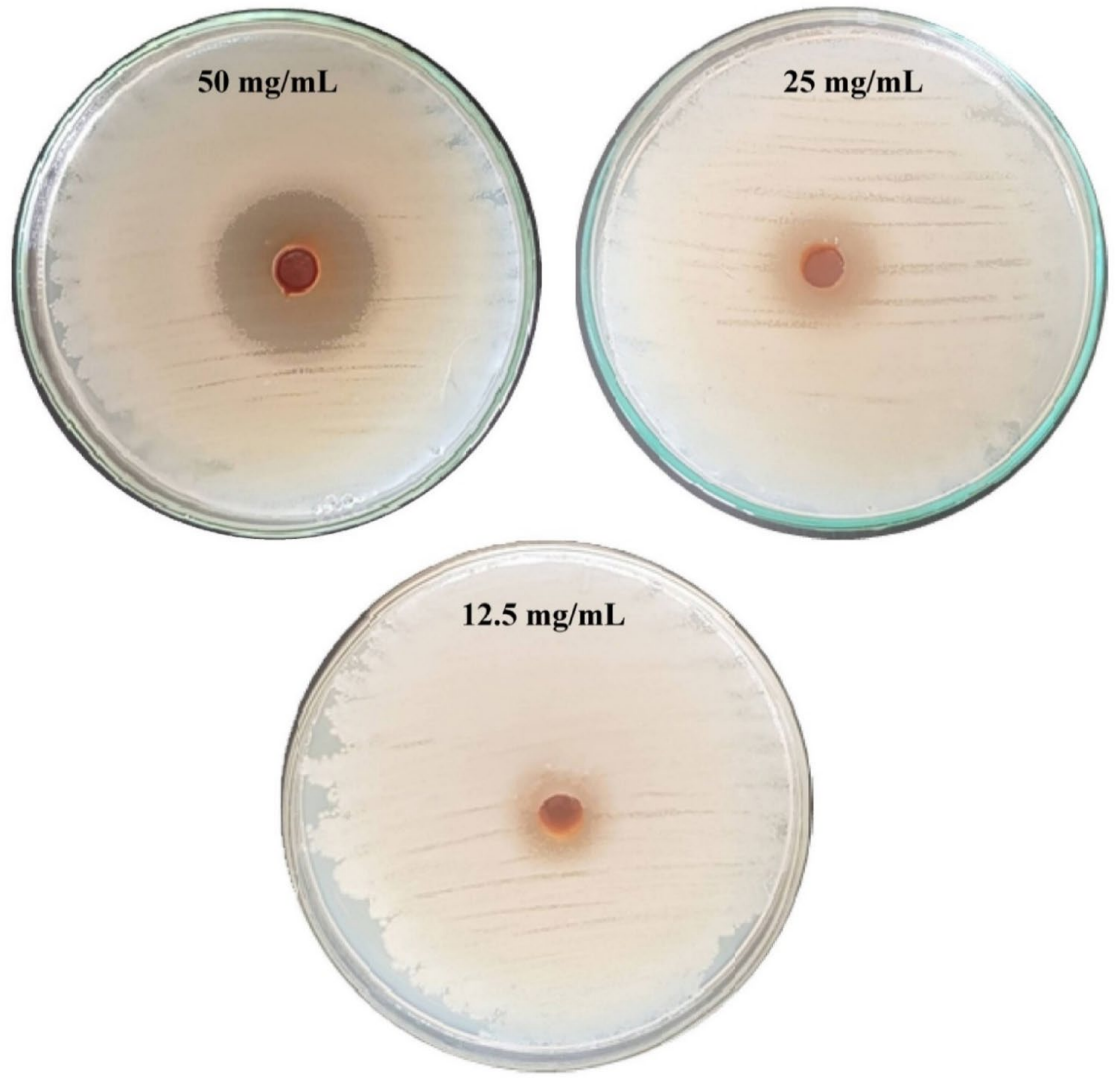
Antibacterial activity of different concentrations of chitosan nanoparticles produced using *Eucalyptus* leaves extract against *Acinetobacter* *baumannii*.

Multi-drug resistant bacteria *Acinetobacter* *baumannii* complex was used to carry out the antibacterial activity tests of CNPs with concentrations of 12.5, 25 and 50 mg/mL. After incubation for 24 h, the inhibition zone diameter created by the well containing CNPs was recorded: 12, 16, 30 mm diameter, respectively. As shown in Fig. 6. The inhibition of bacterial growth increased as CNPs concentrations increased.

### TEM examination of the effect of CNPs on multi-drug resistant *A. baumannii* cells

**Figure 7:**
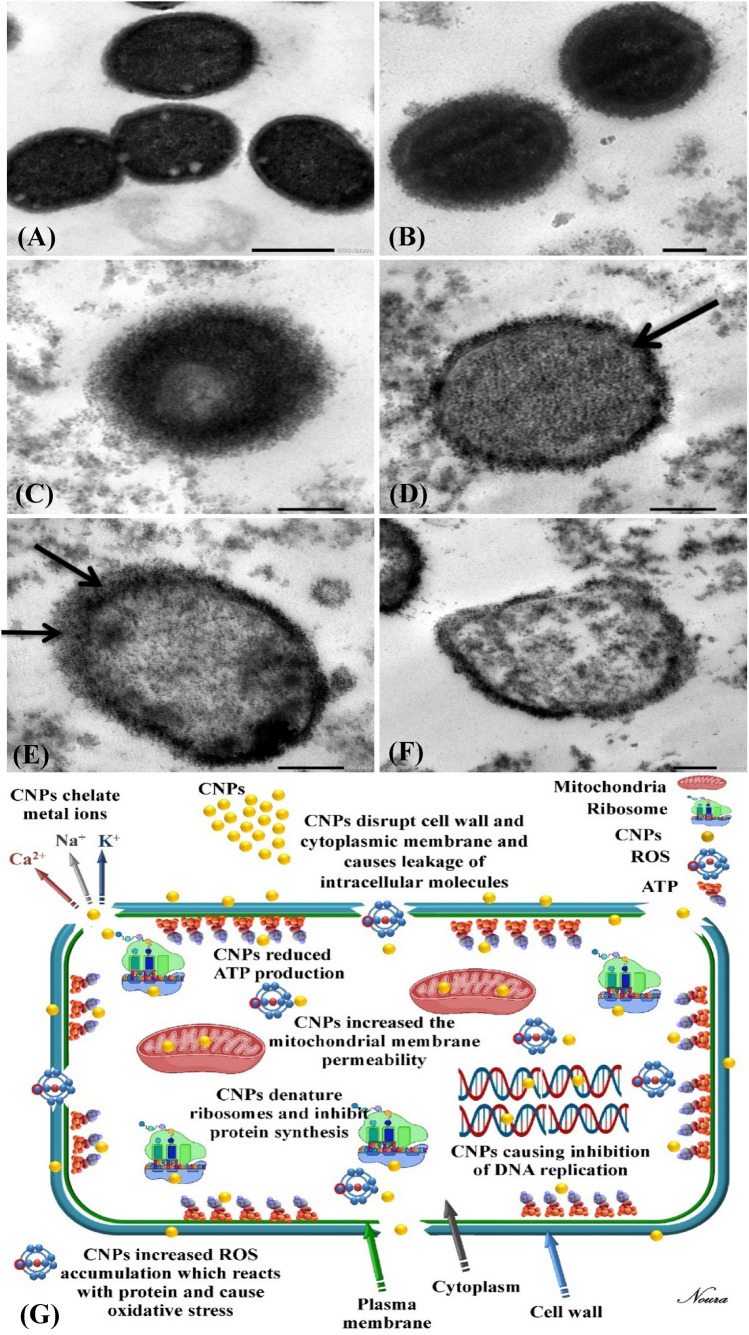
TEM examination of the effect of CNPs on multi-drug resistant *A.* *baumannii* cells: (**A**) cells of untreated bacteria, (**B–F**) cells of bacteria treated with CNPs with different stages in damage and (**G**) mechanisms of antibacterial action of CNPs.

The antibiotic resistance of *A.* *baumannii* complex is becoming increasingly serious. Colistin and Polymyxin, that target the cell membrane, are thought to be the final line of defense against drug-resistant bacteria, but they come with a lot of side effects, and drug resistance to these drugs is increasing gradually^99^. This study tried to control the growth of multi-drug resistant *A.* *baumannii* complex using biosynthesized CNPs. To study the changes in the morphology of *A.* *baumannii* complex cells treated with CNPs. The control (untreated) cells of *A.* *baumannii* complex were represented in Fig. 7A, in which a well-defined cell membrane was seen (the inner and outer membrane envelopes were smooth). The cytoplasmic content of the bacterial cell was regularly distributed. Compared with untreated cells, considerable morphological variations were detected in *A.* *baumannii* complex cells treated with CNPs. As shown in Fig. 7B a great number of CNPs were detected around the bacterial cell membrane. The damage in the cell membrane and the cytoplasm content leaked to the extracellular medium with increases in the periplasmic space (black arrow head) (Fig. 7C–E) the bacterial cells outer membrane is enlarged and evacuated from the plasma membrane. In addition, coagulated material was observed in the cytoplasm. Figure 7F Due to the loss of most cytoplasmic contents from the inner membrane, the outer membrane is enlarged and evacuated, resulting in complete membrane loss; ghost cells^100^.

### Mechanisms of antibacterial action of CNPs

Several studies on chitosan nanoparticles revealed the stronger antibacterial activity of CNPs against Gram-negative and Gram-positive bacteria, Fig. 7G explain the antibacterial actions of CNPs. The presence of amine groups (NH_3_^+^) in glucosamine provides chitosan its polycationic nature, which may be a significant function in its ability to attach with negatively charged surface components of many microorganisms, causing wide variations to the cell surface and subsequent leakage of intracellular substances, resulting in cell death^101^. Chitosan nanoparticles have the properties of chitosan as well as the benefits of nanoparticles. The unique properties of nanoparticles, such as their small size and quantum effects, can offer chitosan nanoparticles with higher capabilities. This is due to the fact that the characteristics of bulk materials stay relatively constant regardless of volume; but, as their size reduces, the percentage of surface atoms increase, creating nanoparticles with some remarkable characteristics^102^. Avadi et al*.*^103^ also mentioned the chitosan nanoparticles higher antibacterial activity than bulk chitosan due to the polycationic chitosan nanoparticles with a higher surface charge density interact with bacteria more effectively than chitosan. For a quantum-size impact, chitosan nanoparticles provide a stronger affinity towards bacteria cells. Chitosan nanoparticles are able to provide significant antimicrobial properties through various mechanisms. 4 mechanisms have been suggested to describe how chitosan attach to the surface of Gram-negative bacteria (either the outer membrane or the cell wall) affecting their antimicrobial activity: (a) Positively charged chitosan interacts with negatively charged residues on the bacterial surface to modify cell permeability through an electrostatic interaction; (b) Chitosan penetrates through the cell membrane and adsorb onto the DNA molecules leading to blocking the transcription of RNA, inhibition of mRNA synthesis and protein synthesis^102,104^; (c) Chitosan inhibits microbial growth by chelating essential nutrients and minerals. Smaller molecules like potassium and phosphate seep out, followed by larger molecules like RNA and DNA etc.^105^. Chandrasekaran^106^ reported that chitosan nanoparticles have metallic ion chelation property which is a possible reason for its antimicrobial action; (d) Chitosan has the ability to create a polymer film on the cell surface, which acts as a barrier to oxygen and prevents nutrients from entering the cell, inhibiting aerobic bacterial growth^107^. Nanoparticles have a greater affinity to produce excess quantities of reactive oxygen species (ROS). Due to the strong oxidation potential, additional ROS produced by nanoparticles can damage biomolecules and organelle components and cause oxidative carbonyl of protein, fat peroxide, DNA/RNA fracture, and destruction of membrane structure, leading to more necrosis, apoptosis or even mutations^108^. Dizaj et al.^109^ described that the antimicrobial activity of nanostructured materials (NSM) includes the creation of reactive oxygen species (ROS) which induces an increase in oxidative stress in microbial cells. Highly elevated amounts of reactive oxygen species (ROS) and other free radicals cause mitochondrial and endoplasmic reticulum disorder, along with severe damages to biomolecules, resulting in genotoxic effects.

## Conclusions

In this study CNPs have been biologically synthesized and characterized, the CNPs obtained have small particle size with a regular spherical shape and positive surface charges, the antibacterial experiment indicated that the CNPs exhibited excellent antibacterial properties against *Acinetobacter* *baumannii* complex. The biologically synthesized CNPs could be suitable for biological applications in medical treatments and food preservation. Nevertheless, CNP's mechanisms of action against bacteria have not yet been fully elucidated. Therefore, investigations on the antibacterial mechanisms of CNPs and toxicological studies are necessary.

## Supplementary Information

Supplementary Figures.

## Data Availability

All data generated or analyzed during this study are included in this article.
