# Supplementary material for: Green synthesis of chitosan nanoparticles, optimization, characterization and antibacterial efficacy against multi drug resistant biofilm-forming Acinetobacter baumannii
Source: Sci Rep. 2022 Nov 18;12:19869. doi: 10.1038/s41598-022-24303-5 (PMC9674591; doi:10.1038/s41598-022-24303-5)
Supplement: Supplementary file 1 — Supplementary Figures. [file 41598_2022_24303_MOESM1_ESM.pdf]

Original Article

Green synthesis of chitosan nanoparticles, optimization, characterization and antibacterial efficacy against multi drug resistant biofilm-forming *Acinetobacter baumannii*

Noura El-Ahmady El-Naggar[1], Alaa M. Shiha[2], Hoda Mahrous[3], A. B. Abeer Mohamed[4]

[1] Department of Bioprocess Development, Genetic Engineering and Biotechnology Research Institute, City of Scientific Research and Technological Applications (SRTA-City), New Borg El-Arab City 21934, Alexandria, Egypt.

[2] Microbial Biotechnology Department, Genetic Engineering and Biotechnology Research Institute, University of Sadat City, Egypt.

[3] Industrial Biotechnology Department, Genetic Engineering and Biotechnology Research Institute, University of Sadat City, Egypt.

[4] Microbial Biotechnology Department, Genetic Engineering and Biotechnology Research Institute, University of Sadat City, Egypt.

Corresponding Author's information

Dr. Noura El-Ahmady Ali El-Naggar

Address:

Bioprocess Development Department,

Genetic Engineering and Biotechnology Research Institute,

City of Scientific Research and Technological Applications,

New Borg El- Arab City, 21934, Alexandria, Egypt

Tel: (002)01003738444 **Fax:** (002)03 4593423

E-mail: nouraalahmady@yahoo.com

Results

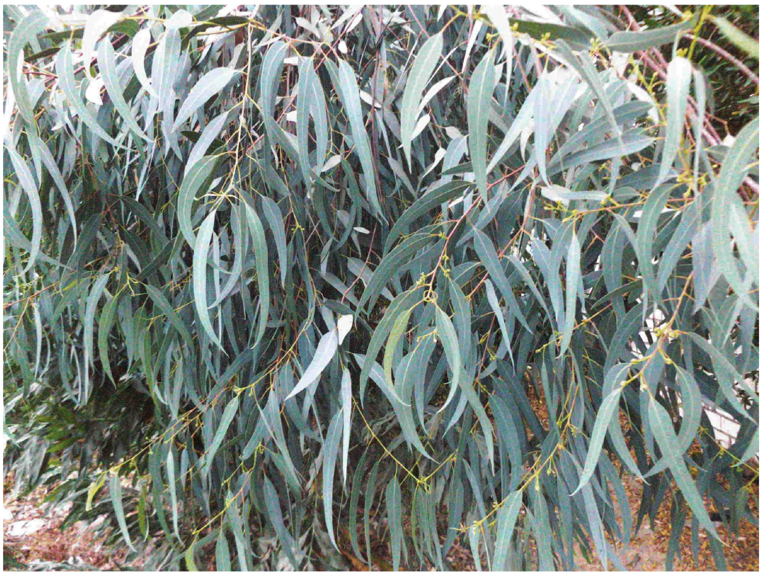

Supplementary Figure S1. *Eucalyptus citriodora* leaves

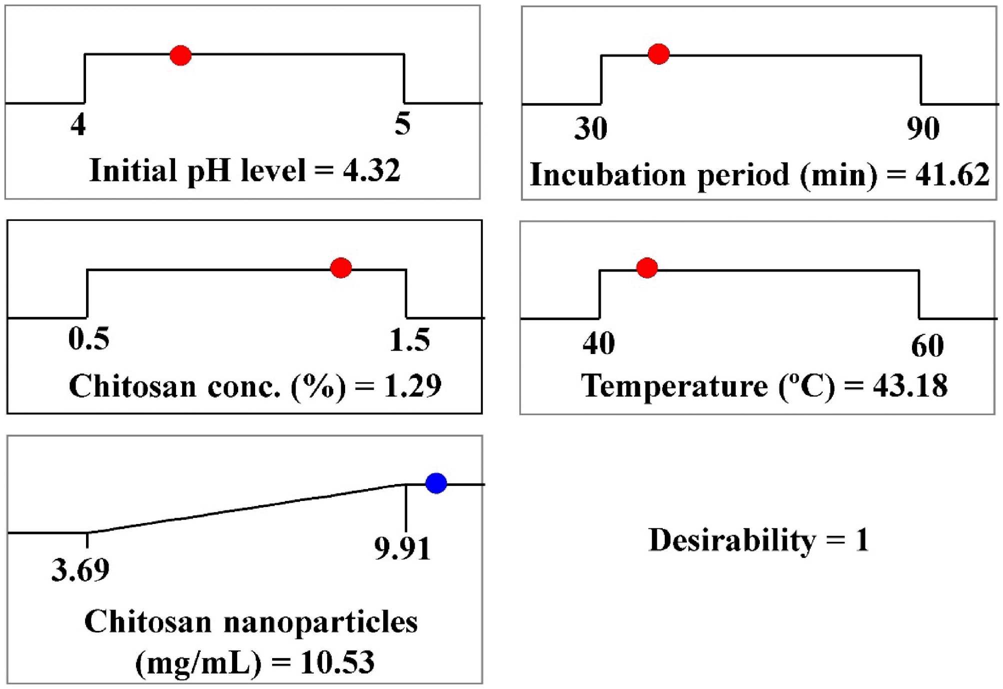

Supplementary Figure S2. The desirability function and the optimum predicted values for the maximum chitosan nanoparticles biosynthesis using *Eucalyptus citriodora* leaves extract.
